# Development and semantic validation of an instrument for the assessment of knowledge and attitudes towards cardiopulmonary resuscitation in adolescents*

**DOI:** 10.17533/udea.iee.v40n1e15

**Published:** 2022-03-30

**Authors:** Yrene Esperanza Urbina-Rojas, Zoila Esperanza Leiton-Espinoza, Angel López-González, Joseba Rabanales-Sotos, Alice Regina Felipe Silva, Jack Roberto Silva Fhon

**Affiliations:** 1 Nurse, Doctor. Email: yurbina@untumbres.edu.pe. Nursing College, Universidad Nacional de Tumbes. Perú Universidad Nacional de Tumbes Nursing College Universidad Nacional de Tumbes Peru yurbina@untumbres.edu.pe; 2 Nurse, Doctor. Email: zeleiton@unitru.edu.pe Nursing College,Universidad Nacional de Trujillo. Perú Universidad Nacional de Trujillo Nursing College Universidad Nacional de Trujillo Peru zeleiton@unitru.edu.pe; 3 Male Nurse, Doctor. Email: angel.lopez@uclm.es Nursing College, Albacete, Universidad de Castilla-La Mancha. España Universidad de Castilla-La Mancha Nursing College Universidad de Castilla-La Mancha Albacete Spain angel.lopez@uclm.es; 4 Male Nurse, Doctor.Emaill: joseba.rabanales@uclm.es Nursing College, Albacete, Universidad de Castilla-La Mancha. España Universidad de Castilla-La Mancha Nursing College Universidad de Castilla-La Mancha Albacete Spain joseba.rabanales@uclm.es; 5 Nurse, Mastering. Email: alice.regina.silva@alumni.usp.br Nursing College, Universidad de Sao Paulo. Brasil Universidad de Castilla-La Mancha Nursing College Universidad de Sao Paulo Spain alice.regina.silva@alumni.usp.br; 6 Male Nurse. Email: betofhon@usp.br Nursing College, Universidad de Sao Paulo. Brasil Universidade de São Paulo Nursing College Universidad de Sao Paulo Brazil betofhon@usp.br

**Keywords:** validation study, cardiopulmonary resuscitation, health knowledge, attitudes and practice, adolescent, nursing methodology research., estudio de validación, reanimación cardiopulmonar, conocimiento, actitudes y prácticas en salud, adolescente, investigación metodológica en enfermería., estudo de validação, reanimação cardiopulmonar, conhecimentos, atitudes e prática em saúde, adolecente, pesquisa metodológica em enfermagem.

## Abstract

**Objective.:**

Develop and semantically validate an instrument to assess the knowledge and attitudes of adolescents towards cardiopulmonary resuscitation (CPR).

**Methods.:**

Validation study of an instrument to evaluate the knowledge and attitudes of adolescents towards CPR, developed in three phases: (i) development of the evaluation instruments by the authors; (ii) content validation performed by 14 expert judges in the area using the content validity index for analysis; and (iii) semantic validation carried out with the participation of 30 adolescents between 11 and 13 years old.

**Results.:**

In the content validation, the questions on CPR knowledge obtained a content validity index (CVI) between 0.92 and 1.00, with a general index of 0.98; and the questions about attitudes obtained an IVC between 0.85 and 1.00, with a general index also of 0.98. Regarding semantic validation, three questions were modified in the knowledge assessment instruments and five in the attitude assessment instrument.

**Conclusion.:**

Semantic and content validation of the instruments studied showed that they are suitable for assessing the knowledge and attitudes of adolescents related to CPR, so their use is recommended in the evaluation of training actions in this population group.

## Resumen

**Objetivo.:**

Desarrollar y validar semánticamente un instrumento para evaluar los conocimientos y las actitudes de adolescentes hacia la resucitación cardiopulmonar (RCP).

**Métodos.:**

Estudio de validación de un instrumento para evaluar los conocimientos y las actitudes de adolescentes hacia la RCP que se desarrolló en tres fases: (i) desarrollo de los instrumentos de evaluación por parte de los autores; (ii) validación de contenido realizada por 14 jueces expertos en el área utilizando el índice de validez de contenido para el análisis; y (iii) validación semántica realizada con la participación de 30 adolescentes, entre los 11 y 13 años.

**Resultados.:**

En la validación de contenido, las preguntas sobre conocimientos de RCP obtuvieron un índice de validez de contenido (IVC) entre 0.92 y 1.00, con índice general de 0.98; y las preguntas sobre actitudes obtuvieron un IVC entre 0.85 a 1.00, con un índice general también de 0.98. En cuanto a la validación semántica, se modificaron tres preguntas en los instrumentos de evaluación de conocimientos y cinco en el instrumento de evaluación de actitudes.

**Conclusión.:**

La validación semántica y de contenido de los instrumentos estudiados mostraron que son adecuados para la valoración de conocimientos y actitudes de los adolescentes relacionados con la RCP, por lo que se recomienda su uso en la evaluación de acciones formativas en este grupo poblacional.

## Resumo

**Objetivo.:**

Desenvolver e validar semanticamente um instrumento para avaliar os conhecimentos e as atitudes de adolescentes para a ressuscitação cardiopulmonar (RCP).

**Métodos.:**

Estudo de validação de um instrumento para avaliar os conhecimentos e as atitudes de adolescentes para a RCP que se desenvolveu em três fases: (i) desenvolvimento dos instrumentos de avaliação por parte dos autores; (ii) validação de conteúdo realizada por 14 juízes especialistas na área utilizando o índice de validez de conteúdo para a análise; e (iii) validação semântica realizada com a participação de 30 adolescentes de entre 11 e 13 anos.

**Resultados.:**

Na validação de conteúdo, as perguntas sobre conhecimentos de RCP obtiveram um índice de validez de conteúdo (IVC) entre 0.92 e 1.00, com índice geral de 0.98; e as perguntas sobre atitudes obtiveram um IVC entre 0.85 a 1.00, com um índice geral também de 0.98. Enquanto à validação semântica, se modificaram três perguntas nos instrumentos de avaliação de conhecimentos e cinco no instrumento de avaliação de atitudes.

**Conclusão.:**

A validação semântica e de conteúdo dos instrumentos estudados mostraram que são adequados para a valoração de conhecimentos e atitudes dos adolescentes relacionados com a RCP, por isso se recomenda seu uso na avaliação de ações formativas neste grupo populacional.

## Introduction

Out-of-hospital cardiac arrest is the third leading cause of death in the world, it represents one of the main global health problems, constituting a public health problem.(1) Published data indicate that the annual incidence of out-of-hospital cardiac arrest in the European countries ranges from 67 to 170 per 100,000 population, with European citizens initiating resuscitation in 58% of cases.(2) Immediate initiation of cardiopulmonary resuscitation (CPR) by a citizen improves survival from cardiac arrest out-of-hospital and it is estimated that the chances of survival can be doubled or tripled. Therefore, designing strategies to teach CPR will increase the individual's chances of survival.(3)

The World Health Organization endorsed the Kids Save Lives statement, recognizing that teaching CPR to children and adolescents is an effective way to increase care for people experiencing out-of-hospital cardiac arrest. This learning, moreover, has a multiplier effect, which supports its inclusion in the school curriculum.(4) The International Liaison Committee on Resuscitation recommends teaching CPR from children and having evaluation instruments that allow estimating perceived self-efficacy or judgments of each individual on their abilities.(5) The school environment is ideal for promoting knowledge and learning basic CPR techniques. It is known that children under 13 years of age have difficulties in performing some of the basic resuscitation techniques due to their physical characteristics, however, they are able to learn and remember these and other theoretical aspects related to the initial care provided to patients in cardiac arrest. (CA), promoting knowledge and skills step by step, according to age and stage of training^.^ (6) There are various programs, methods and teaching materials proposed for teaching CPR from an early age. It has been observed that children and adolescents are capable of assimilating the knowledge and skills necessary to perform CPR. Also, they are able to transmit them among their own family and friends. However, the best training method for each population group has yet to be defined. (3,7)

In CPR training, the instruments for the evaluation of knowledge acquired by the participants must be validated in terms of content, appearance, criteria and construct, to be reliable.(8) The evaluation of the content of an instrument is considered a fundamental step in this design. It represents a beginning in the association mechanisms of abstract concepts as indicators that can be observed and measured.(9)

Semantic validation indicates whether it is well constructed from the communicative point of view, that is, it adequately shows its intention and purpose.(10) One of the greatest challenges in the development of assessment instruments aimed at children and adolescents involves the need of the specialized use of words and syntactic structures, common in the academic field, but foreign to them. (11) Mistakes in the decoding of information make difficult for children and adolescents to read comprehension,(12) so in this sense, semantic validation will allow a readjustment of terms so that adolescents understand the questions in their entire context.

Attitudes refer to people's perceptions of their ability to perform certain tasks.(13) They allow determining aspects of fear and anxiety to face new and/or stressful situations. Attitudes involve motivation and cognitive resources, and the actions needed to achieve certain goals are more likely to occur when high self-efficacy is perceived.(8) There are different models and instruments that measure the knowledge and emotional response in health sciences professionals and students to CPR training, (8,14,15) but there are no validated scales that assess whether the adolescent population feels capable of use their knowledge and skills in a PC situation after a CPR training process. In this sense, the objective of the present study was to construct and semantically validate instruments to evaluate the knowledge and attitudes of adolescents related to basic CPR.

## Methods

This is a methodological study that is part of the research entitled "Basic cardiopulmonary resuscitation training project in children and adolescents of a public educational institution. Tumbes, Peru, 2019”. This phase of the study was carried out in three stages: (a) development of the evaluation instruments; (b) content validation by expert judges; and (c) semantic validation by the target population.

The ad hoc instrument to assess CPR knowledge was created based on the 2020 Basic CPR Guidelines and Protocols of the American Heart Association (AHA) and the 2021 European Resuscitation Council (ERC).(16,17) The questions evaluated only those techniques that are considered safe to perform in the current pandemic environment following the CPR Guidelines in a pandemic situation. (18) It was made up of eight questions with four answer options, of which only one was correct, and the participants could choose one of the options. The CPR attitude assessment instrument is made up of 11 questions with dichotomous answers (yes and no) and aims to identify the attitudes of adolescents in relation to the care they would offer to a CA. The items of this instrument are based on what was proposed by Navalpotro and Torre. (14)

Content validation was used in the study in order to determine the representativeness of the items of the proposed instruments. The judgment of specialists in the area was available,(19) which allowed for an effective exploration of the requirements to measure the phenomenon under investigation. A committee of expert judges in the area of basic CPR was formed. Fifteen specialists were invited, chosen for convenience due to their professional experience and postgraduate studies in the area of urgencies and emergencies. Of those invited, 14 agreed to participate in the research. The instruments were sent by e-mail and they were given a period of 15 days to return them with their suggestions. (13)

The content validity index (CVI) was used to analyze the responses of the judges. This index allows to analyze the instruments as a whole, and each item individually using a Likert-type scale from 1 to 4. Its purpose was to measure the proportion of specialists who agreed with the content presented. (13) The study met the number minimum number of specialists recommended by the literature. (17) In addition, values of at least 0.78 were considered for the validity of each item of the instruments, and of at least 0.90 for the validity of the instruments as a whole.(8,19) 

The semantic validation was carried out through interviews with a structured instrument to 30 adolescents of both sexes, between January and February 2021. A convenience sample was used, and the inclusion criteria were that the adolescents had to be between 11 and 13 years old, age considered by the WHO as early adolescence, and similar to that of the students in which the instruments will be applied and an adequate level of reading comprehension in Spanish. Three groups of 10 participants were formed for each age group (11, 12 and 13 years).

To collect this information, meetings were scheduled with adolescents, carried out through Google Meet® or in person by previously trained nurses, lasting 30 minutes. During the interview, the instrument was read to the adolescent to identify if he understood the wording of the question and the response options. When the participant reported that he did not understand or did not know the word or its meaning, he was asked to say what he understood and synonyms were dictated that could replace the unknown word.

This study respected the ethical principles of the Helsinki Convention. To participate in the study, the adolescents had to agree and present the signed authorization of their parents and/or guardians. The research project was approved by the Research Ethics Committee of the National University of Tumbes with number 001-2021/CEI-UNTUMBES.

## Results

The committee of expert judges was made up of 13 nurses and one male nurse. The average age was 53 years, with an average time of professional practice of 27.2 years. Regarding degrees, 71.4% had a master's degree and 14.3% were specialists in emergency and intensive care, the remaining 14.3% were graduates in nursing without a specialty. Regarding workplaces, 50% worked in intensive care units, 50% in emergency services. In addition to their professional practice, 78.6% of the judges were professors in undergraduate, specialty and/or postgraduate programs at universities in Peru.

Regarding the content validity of the instrument to evaluate knowledge related to CPR, after the evaluation carried out by the experts, of the eight questions of the instrument, two of these obtained an CVI applied to questions (CVI-I) > 0.92, and six of them obtained a score of 1. (Table 1) The general CVI obtained a score of 0.98.

**Table 1:** Content Validation Index of the instrument developed to assess knowledge about Basic Cardiopulmonary Resuscitation in adolescents, obtained through the evaluation of specialist. Peru, 2021

Questions	CVI-1
1 what is a heart arrest?	1
a) The heart stops beating and the person does not breathe.	
b) The heart slowly stops beating.	
c) Absence of breathing.	
d) Sudden loss of consciousness.	
2. What is the first step to be taken in front of a person with loss of consciousness?	1
a) Request help from the health service by calling 116*.	
b) Extend the neck backwards so that the air enters.	
c) Move the unconscious person to wake up.	
d) Be by the person's side until the ambulance arrives.	
3. What should I say when I telephone the emergency service, for example, the fire department 116?	1
a) I inform that the person does not respond, is not breathing and where I am.	
b) I shout reporting my data asking for help and hang up immediately.	
c) I ask another person to pass on the information about what is happening with the person.	
d) I scream and ask someone else to pass the information to the health personnel.	
4. While the ambulance arrives, how many compressions should I perform	1
a) 30 compressions per minute.	
b) 15 compressions per minute.	
c) 100-120 compressions per minute.	
d) Those necessary until the ambulance arrives.	
5. If after doing the compressions in the center of the person's chest I CHECK, HE (or SHE) IS BREATHING, what should I do?	0,92
a) I sit him down and give him water.	
b) I put him on its side.	
c) I tell him what happened.	
d) I take him to a cooler place.	0,92
6. If after some time performing compressions in the center of the person's chest, I verify that he/she is still unconscious and not breathing, what should I do?	
a) I will continue with compressions until help arrives.	
b) I ask someone to help and I start filming.	
c) I ask someone else to do the compressions while I rest.	
d) I continue compressions, even if I feel very tired.	
7. Why should compressions be given to the center of the chest in the person in cardiac arrest?	1
a) Because with each compression it stimulates the heart and blood reaches the entire body.	
b) For him to wake up, walk and go home.	
c) Because the air enters the lungs better.	
d) Because the person may be choking and thus the foreign body may come out.	
8. What should we do when the ambulance arrives?	1
a) I retire and go home.	
b) I start filming with my cell phone.	
c) Explain to him what has been done until his arrival.	
d) Acompany he person to the hospital.	

CVI-1=Content Validation Index applied to the questions. * Emergency phone number - Firefighters of Peru

With respect to the instrument for assessing attitudes of adolescents towards a witnessed CA and CPR, two questions obtained an CVI-l >0.85, and nine of them obtained a score of 1. The overall CVI score was 0.98 (Table 2).

**Table 2:** Content Validation Index of the instrument developed to assess attitudes about Basic Cardiopulmonary Resuscitation in adolescents, obtained through the evaluation of specialists. Peru, 2021

Questions	CVI-I
1. I feel capable and prepared to react to an emergency situation.	1
2. I am able to remain calm when faced with a person who has fainted and is NOT breathing.	1
3. I feel capable of making decisions when I am faced with a person who has fainted and is NOT breathing.	1
4. I am able to quickly call an emergency system, for example, the fire department (116), when I find a person who has fainted and is NOT breathing.	1
5. I feel able to report the details to the telephone operator of the emergency system, for example, the fire	1
6. I feel able to open the airway in an unconscious person.	0.92
7. I feel able to perform center chest compressions on an unknown person in cardiac arrest.	1
8. I feel able to perform center chest compressions on a close family member in cardiac arrest.	0.85
9. I feel capable of informing the health professionals of the procedure performed on the person in cardiac arrest. 1	1
10. I feel able to LEARN Cardiopulmonary Resuscitation because with it I can save lives.	1
11. I feel able to PERFORM Cardiopulmonary Resuscitation because with it I can save lives.	1

CVI-I Content Validation index applied to questions

During the semantic validation, the suggestions of the participants generated modifications in some terms of the instruments proposed by the experts in order to improve understanding, keeping the focus on the evaluation of the knowledge and attitudes of adolescents during CPR. Regarding the modifications made in the knowledge evaluation instrument (Table 3), the answer "c" of question 1 was modified, substituting the word "absence" for "lack". Answer "d" to the same question was also changed, replacing the word "sudden." with “abrupt” The statement of question 3 was initially "What should I say when I call the Emergency Service, for example, the fire department 116?" was changed to What should I say when I call the Emergency number, for example the firefighters 116? Initially, the statement of question 5 was "If after performing the compressions in the center of the person’s chest, I CHECK THAT THEY ARE BREATHING, what should I do?" and was replaced with "If after performing compressions in the center of the person's chest, I CONFIRM THEY ARE BREATHING, what should I do?"

**Table 3:** Semantic validation of the Instrument developed to assess knowledge about Basic Cardiopulmonary Resuscitation performed by adolescents. Peru, 2021

1- What is a heart arrest?
c) **lack** of breathing.
d) **abrupt** loss of conciousness.
3. ¿ What should I say when I call the emergency **number,** for example, the fire department 116?
5If after doing the compressions in the center of the person's chest **I CONFIRM THEY ARE BREATHING**

Note: In bold the modified words

In relation to the attitude evaluation instrument, questions one, four and five were modified as follows (Table 4): Question 1, initial statement “I feel able and I am prepared to react to an emergency situation”, final statement, “I feel able and I am prepared to react to an emergency in which life is in danger”; Question 4, initial statement "I feel able of quickly calling an emergency system, for example, the fire department (116), when I find a person who has fainted and is NOT breathing", final statement, "I feel able of quickly call an emergency number, for example, the fire department (116), when I find a person who has fainted and is NOT breathing“; Question 5, initial statement "I feel able to report the details to the telephone operator of the emergency system, for example, to the firemen, in a calm way", final statement "I feel capable of reporting the details to the emergency telephone operator in a calm way”. In addition, the acronym "CPR" was added after the term cardiopulmonary resuscitation in questions 10 and 11.

**Table 4:** Semantic validation of the instrument developed to assess attitudes related to Basic Cardiopulmonary Resuscitation in adolescents. Peru, 2021

1. I feel able and prepared to react to an emergency **in which life is in danger**.
4**. I feel able to quickly call an emergency number, for example, the fire department (116), when I find a person who has fainted and is NOT breathing**.
5. I feel able to calmly report details to the emergency operator.
10. I feel able to LEARN Cardiopulmonary Resuscitation (CPR) because with it I can save lives.
11. I feel able to PERFORM Cardiopulmonary Resuscitation (CPR) because with it I can save lives.
Note: in bold the modified words

## Discussion

In this study, the content and semantic validity of two instruments developed to determine the knowledge and attitudes of adolescents related to CPR after receiving standardized training were evaluated.

Unlike the study, aimed at adolescents, the levels of competence in the actions that must be carried out during a resuscitation in the hospital or in medicalized ambulances are evaluated in professionals and students of Health Sciences, for instance, the identification of CA by means of carotid pulse palpation, heart rate monitoring, use of a defibrillator, knowledge of drugs, airway instrumentation, among other procedures^.^ (8,14,15,19-21)

The school teaches and develops health promotion and prevention among students. It is important that students learn to identify and care for cardiac arrest^,^ (22) and this training should adequately measure their knowledge and emotional response.(8,14,15) The study verified that all the elements of the instrument proposed to evaluate that the CPR knowledge was adequate and pertinent, all of them individually and collectively obtained high rates of CVI-1 and CVI, respectively, however, some observations were noted by the evaluators and adapted according to suggestions, which coincides with what was reported by Lima *et al*.,(23) in that these adaptations are a fundamental step for the completion of the materials used in the health education activities. The research involved a group of professionals with an affinity for the subject in the content validation, this being a favorable aspect in the results obtained and coinciding with Meneguin *et al,* who refer that the group of professionals presents credibility and a favorable aspect, since it brings together various specialized knowledge on the subject addressed.(24)

The validity of the elements of the instrument to assess attitudes were also adequate and relevant, supported by high CVI-1 and CVI values. Some terms not understood were also reported by adolescents, which were adapted as long as they were not specific and irreplaceable terms in the subject under study and citing again Mielli *et al.* (15) and Broomfield *et al.*,(21) indicate a deficit of knowledge and not understanding. It can be affirmed that the questions asked to assess knowledge, adapted from instruments aimed at health sciences professionals and students,(14) are valid.

Various authors indicate that the level of comprehension of the questions increases after the training intervention, with the doubts being generated from content and not from comprehension.(15,20) The semantic validation of the instruments indicates that they are well constructed from the point of view of communicative, that is, they adequately show their intention and purpose,(10) however, some observations were noted by the adolescents and adapted according to suggestions for understanding the questions in their entire context. Several studies have measured the self-efficacy or self-confidence of professionals and/or students of health sciences regarding their CPR skills, stating that both increase with the experience studied and with the re-accreditation of competence through periodic training activities. (8,14) which is also applicable to the population under study.

As an application in practice, this research provides a useful tool for learning assessment and self-assessment, and a beginning to be able to develop other CPR self-efficacy scales.

This study has several limitations. In the first place, we face the difficulty of locating expert judges with lines of research in CPR in Peru, with at least a master's degree. A second limitation was the adolescents' difficulty in understanding the specific vocabulary used in CPR. Finally, indicate the limitation of having to hold meetings with adolescents over the internet due to confinement due to the COVID-19 pandemic. The future lines of research are aimed at carrying out the psychometric validation of the instruments provided.

## Conclusion

The semantic and content validation of the instruments studied showed that they are suitable for assessing the knowledge and attitudes of adolescents related to CPR, so their use is recommended in the evaluation of training actions in this population group. By using these instruments during the evaluation of cardiopulmonary resuscitation training, the assistance provided to the victim of a CA will benefit from the standardization of the intervention received.
